# Ethanol sensitivity: a central role for CREB transcription regulation in the cerebellum

**DOI:** 10.1186/1471-2164-7-308

**Published:** 2006-12-05

**Authors:** George K Acquaah-Mensah, Vikas Misra, Shyam Biswal

**Affiliations:** 1 Department of Pharmaceutical Sciences, School of Pharmacy-Worcester, Massachusetts College of Pharmacy and Health Sciences, 19 Foster Street, Worcester MA 01608-1715, USA; 2 Department of Environmental Health Sciences, Bloomberg School of Public Health, Johns Hopkins University, 615 North Wolfe St., Baltimore MD 21205, USA

## Abstract

**Background:**

Lowered sensitivity to the effects of ethanol increases the risk of developing alcoholism. Inbred mouse strains have been useful for the study of the genetic basis of various drug addiction-related phenotypes. Inbred Long-Sleep (ILS) and Inbred Short-Sleep (ISS) mice differentially express a number of genes thought to be implicated in sensitivity to the effects of ethanol. Concomitantly, there is evidence for a mediating role of cAMP/PKA/CREB signalling in aspects of  alcoholism modelled in animals. In this report, the extent to which CREB signalling impacts the differential expression of genes in ILS and ISS mouse cerebella is examined.

**Results:**

A training dataset for Machine Learning (ML) and Exploratory Data Analyses (EDA) was generated from promoter region sequences of a set of genes known to be targets of CREB transcription regulation and a set of genes whose transcription regulations are potentially CREB-independent. For each promoter sequence, a vector of size 132, with elements characterizing nucleotide composition features was generated. Genes whose expressions have been previously determined to be increased in ILS or ISS cerebella were identified, and their CREB regulation status predicted using the ML scheme C4.5. The C4.5 learning scheme was used because, of four ML schemes evaluated, it had the lowest predicted error rate. On an independent evaluation set of 21 genes of known CREB regulation status, C4.5 correctly classified 81% of instances with F-measures of 0.87 and 0.67 respectively for the CREB-regulated and CREB-independent classes. Additionally, six out of eight genes previously determined by two independent microarray platforms to be up-regulated in the ILS or ISS cerebellum were predicted by C4.5 to be transcriptionally regulated by CREB. Furthermore, 64% and 52% of a cross-section of other up-regulated cerebellar genes in ILS and ISS mice, respectively, were deemed to be CREB-regulated.

**Conclusion:**

These observations collectively suggest that ethanol sensitivity, as it relates to the cerebellum, may be associated with CREB transcription activity.

## Background

Animal models have facilitated the investigation of the mechanisms of several diseases. For drug addiction in particular, inbred mouse strains have proved to be invaluable [1,2], and have facilitated the mapping of aspects of addiction-related behaviour to specific genetic loci. Inbred Long Sleep (ILS) and Inbred Short Sleep (ISS) mice, for instance, present many contrasts with respect to a number of alcoholism related phenotypes [3-6]. They have been widely used to model ethanol sensitivity [7,8]. Ethanol sensitivity has a genetic basis [9], the comprehensive workings of which remain elusive. Consequently, a comparison of relevant brain region transcriptomes of ILS and ISS mice has the potential of revealing unique patterns of gene expression [10] that could be relevant to the mechanisms of alcoholism.

The cerebellum has long been almost exclusively associated with balance and motor co-ordination. It has relatively recently been found to be more involved with cognition than previously thought [11]. During neurodevelopment, the cerebellum is especially susceptible to ethanol toxicity [12]. Studies indicate a role for activation of the cerebellum in alcoholism. A Functional Magnetic Resonance Imaging study has indicated that ethanol odour-induced craving in untreated recently abstinent male alcoholics involves activation of the cerebellum along with the subcortical-limbic region of the right amygdala/hippocampal area [13]. Positron Emission Tomography studies in drug addiction similarly indicate a role for cerebellar activation [14,15]. The identification of specific pathways contributing to alcoholism-related events in the cerebellum would, therefore, be important.

The phosphoinositide (PI) and cyclic adenosine 3',5'-monophosphate (cAMP) signalling pathways have long been thought to be important in the development of ethanol dependence and tolerance [16]. There are several pieces of evidence suggesting a role for the cAMP/protein kinase A (PKA)/cAMP-response-element-binding protein (CREB) signalling pathway in addiction, even though they do not necessarily involve the cerebellum: Alcohol preferring (P) rats have lower levels of CREB and the transcriptionally-active phospho-CREB in the medial amygdala and central amygdala (CeA) than non-preferring (NP) rats [17]. Ethanol administration (or PKA activator [Sp-cAMP] administration into the CeA) increases CREB function in the CeA of P (but not NP) rats. Also, 24 hours following a single intra-peritoneal 2 mg/kg ethanol dose to C57BL/6J mice, there is long-term potentiation of GABA synaptic transmission at Ventral Tegmental Area dopaminergic neurons, via a cAMP-PKA-dependent mechanism [18]. One mechanism by which ethanol increases CREB levels involves inhibition of adenosine reuptake which results in increases in extracellular adenosine and activation of the adenosine A2 receptor, leading to increases in cAMP levels [19]. The ethanol-induced increase in CRE-mediated gene transcription requires PKA and involves an adenosine receptor-dependent phase and a later adenosine receptor-independent phase [20].

The emergence of high throughput data has facilitated the study of patterns of transcription. Machine Learning (ML) is one such avenue for mining such data [21]. It concentrates on methods for computer programs to improve their performance (i.e. modifying behaviour) by learning from previous data examples. ML is useful for the purpose of class prediction. During the learning process, structural patterns in the "training set" are established; these then constitute the basis upon which predictions are made when presented with data of unknown classification ("test set").

In the current studies, genes found to be differentially expressed in the cerebella of ILS and ISS mice [22] were examined to identify the extent to which CREB transcription regulates addiction mechanisms in the cerebellum. Nucleotide sequences of the promoter regions of various genes were analyzed to generate the data used for ML. The Composition, Transitions, and Distributions [23] of individual nucleotide bases as well as groups of nucleotide bases (Table 1), along with the presence and relative positions of specific *cis *elements were the basis on which genes were classified as being either transcriptionally CREB regulated or otherwise. The results reveal a strong pattern, in the cerebellum, of CREB regulation among genes differentially expressed between ILS and ISS mice.

## Results

Four ML schemes were evaluated: a Decision Tree (J48, an implementation of the C4.5 algorithm), a Support Vector Machine (SVM), a Naïve Bayes classifier (NN) and a Multi-layer Perceptron (MLP). Two alternate models for ML were tested in this study, using a dataset of 46 instances and two classes. These were:

• -a two-class model with classifications: "CREB-regulated" and "NOT CREB-regulated", and

• -a three-class model with a third classification "Nrf2-regulated" [24]

**Figure 1:**
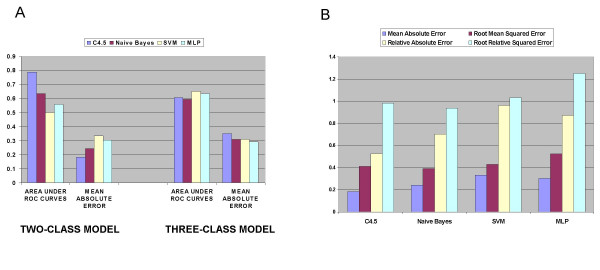
**Learning Scheme Accuracy and Error Rates**. Accuracy and error rates for learning on a two-class and a three-class model (defined in the Methods section), using the Leave-one-out Cross Validation technique. A) A comparison of accuracy and error rates for learning on the two-class model versus the three-class model. The two-class model yields the higher area under the ROC curve and the lower Mean Absolute Errors for the C4.5 and the Naïve Bayes classifiers. B) A depiction of the error rates determined using the two-class model. The C4.5 scheme has the lowest Mean Absolute Error and Relative Absolute Error.

Nrf2 (NF-E2-related factor 2), the primary transcription factor that binds the Antioxidant Response Element (ARE), was selected because, like CREB, Nrf2 is a ubiquitous transcription factor. Secondly, it has a requirement for CREB Binding Protein for enhanced transcription activity [25]. Using the leave-one-out cross-validation technique, the two-class model had lower Mean Absolute Error rates for all learning schemes explored than the three-class model (Figure 1A). Also, of the four schemes and two models evaluated, the area under the Receiver Operating Characteristic (ROC) curve, a measure of test accuracy, was highest for the C4.5 scheme under the two-class model (Figure 1A).

**Figure 2:**
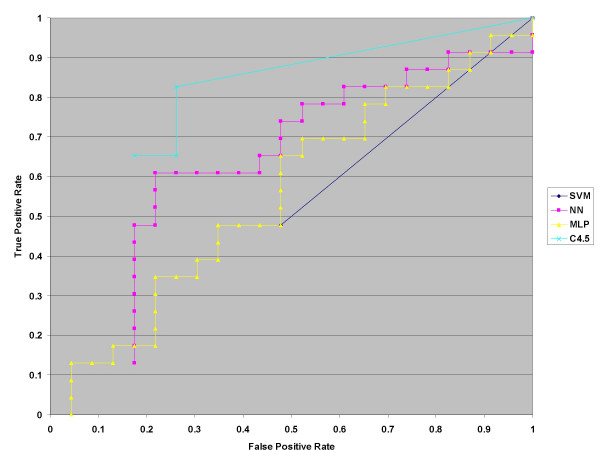
**Learning Scheme ROC Curves**. Receiver Operating Characteristic (ROC) curve for learning schemes using the two-class model and the Leave-one-out Cross Validation technique. The C4.5 test is closest to the left-hand border and the top-border of the ROC space, and therefore the most accurate of the schemes.

Of the four ML schemes, using the leave-one-out cross-validation technique and the two-class model, the C4.5 Decision Tree algorithm had the lowest overall predicted error rate (Figure 1B; Table 2). Its ROC curve was closest to the left-hand border and the top border of the ROC space (Figure 2 and Additional File 1), indicating that it had the most optimal trade-off between sensitivity and specificity among the four schemes evaluated. It also had the highest area under the ROC curve (Table 3). The C4.5 Decision Tree algorithm [26] works top-down, seeking at each stage an attribute that best separates the classes. The attribute with the greatest *information gain *is chosen. It then recursively processes the sub-problems resulting from the split until the *information *either reaches a maximum or is zero. The information measure (*entropy*) is calculated thus:

Entropy (p_1_, p_2_, .... p_n_) = -p_1_**log_2_**p_1_-p_2_**log_2_**p_2_....-p_n_**log_2_**p_n_

where p_1_, p_2_, .... p_n _are fractions representing the data distribution at a node (attribute) and sum up to 1.

The two-class model was also used to test an independent dataset generated from 21 genes of known CREB regulation status. C4.5 correctly classified 81% of instances (Table 4) with F-measures of 0.87 and 0.67 respectively for the classes "CREB-regulated" and "NOT CREB-regulated" respectively. The F-measure is the harmonic mean of Precision and Sensitivity and can be used as a single measure of a test's performance:

F-measure = (2 * Precision * Sensitivity)/(Precision + Sensitivity)

where Precision = True Positives/(True Positives + False Positives)

*Sensitivity (or Recall) *is a measure of the probability that the test would reject a false null hypothesis:

Sensitivity = True Positives/(True Positives + False Negatives)

Additionally, using the two-class model, three out of four genes determined by two independent microarray platforms to be up-regulated in the ILS cerebellum [22] were determined by C4.5 to be transcriptionally CREB-regulated (Table 5). The platforms were the Affymetrix (Santa Clara, CA) platform Mouse Expression Set 430 (MOE430) and the cDNA arrays NIA15K manufactured at the University of Colorado's School of Medicine. Similarly, three out of four genes up-regulated by both platforms in the ISS cerebellum were deemed CREB-regulated (Table 6). Furthermore, 64% and 52% of a cross-section of other up-regulated cerebellar genes in ILS and ISS mice, respectively (as per the MOE430 platform), were deemed CREB-regulated.

## Discussion

Lowered sensitivity to the effects of ethanol increases the risk of developing alcoholism. Differing sensitivities to ethanol is, at least in part, attributable to heredity [9], and inbred mouse strains have facilitated the investigation of this complex behavioral phenomenon. In studying CREB's gene regulating activity in ethanol sensitivity, a set of differentially expressed genes in the ILS/ISS mouse model of ethanol sensitivity were examined. The two-class model had lower error rates than the three-class model (Figure 1A). This is probably due to the inherent difficulty of distinguishing between the classifications "CREB-regulated" and "Nrf2-regulated". Indeed the case can be made that Nrf2 genes are dependent on CREB for enhanced transcription activity [25]. The complexity of the machinery for transcription makes the two-class model the preferred model for this study.

Properties of stretches of nucleotides can impact their affinity for specific transcription factors; this principle can be exploited for its therapeutic promise [27]. A central premise of this observation is the fact that the characteristics of individual nucleotide bases in any such oligonucleotide contribute to its structure and function [28]. As an example, hydrogen-bonded base pairs help determine the structure and function of nucleic acids. Strength of hydrogen bonding and other nucleotide base classifications used in generating the characteristics of each gene's promoter sequence for this ML study have been outlined in Table 1.

Of the four learning schemes evaluated using the two-class model, C4.5 was the most consistent performer, having the lowest overall error rates (Figure 1B), and the highest accuracy (Figure 2; Table 3), area under the ROC curves being measures of test accuracy. Because of variability between independent evaluation sets, performance evaluations based on evaluation sets are only instructive when such evaluation sets are large in size. Since the evaluation set used consisted of only 21 instances, the cross-validation techniques are better indicators of each learning scheme's performance. The Ten-fold Cross Validation technique is a standard way for predicting the error rate of a learning scheme [29,30]. When applying this technique, an average value is obtained for ten different sets of the re-organized data such that in each case, 90% of the data is used for training and 10% used for testing. The leave-one-out technique is, in essence, an *n*-fold cross-validation technique (*n *being the number of instances in the dataset) and, for a small dataset, a good predictor of a scheme's performance on an independent dataset. In this study, 81% of genes of known classification used as an evaluation set were correctly classified by C4.5 (Table 4), with F-measures of 0.87 and 0.67 respectively for the classes "CREB-regulated" and "NOT CREB-regulated" respectively.

**Figure 3:**
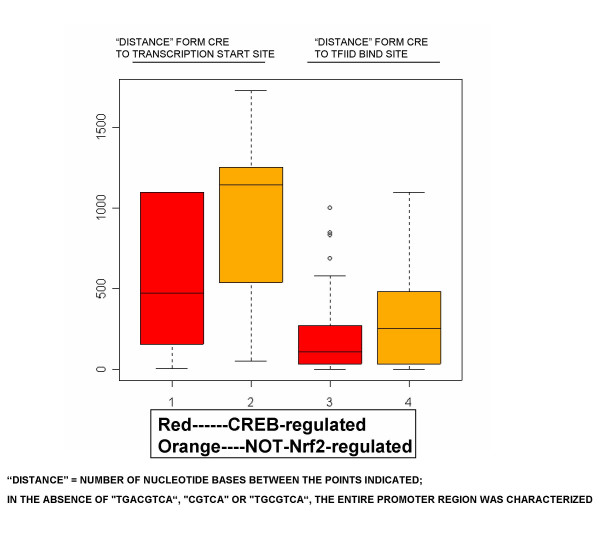
**Promoter Region Distance Metrics**. Boxplots depicting the relative positions of the CREB Response Element (CRE), the Transcription Start Site (TSS), and the Transcription Factor IID binding site. Promoter regions of genes regulated by CREB were more likely to have CREs. In the absence of the CRE ("TGACGTCA", "CGTCA" or "TGCGTCA") in a gene's promoter, the entire promoter sequence was characterized, resulting in longer "distances" for those transcriptionally CREB-independent genes.

The stretch of nucleotides between the cAMP Response Element (CRE) and the Transcription Start Site (TSS) and the stretch between the CRE and the Transcription Factor II D (TFIID) bind site were identified as important determinants of a gene's CREB regulation status (Figure 3). Two types of CRE with different affinities for the transcription factor CREB have been reported. One class containing the symmetrical TGACGTCA site shows a high binding affinity for CREB; the other type has asymmetric and weak binding sites ("CGTCA") [31]. The TATA-binding protein (TBP) and TBP-associated factors (TAFs) constitute the TFIID complex. The TFIID complex is a major component of the general RNA polymerase II (RNAP II) transcription machinery with intrinsic sequence-specific DNA-binding activity [32]. The binding of TFIID to a gene's core promoter region is an important rate-limiting step in the assembly of the transcription initiation complex. With the notable exception of the stretch between the CRE and the TFIID bind site, CREB target promoter regions have relatively high levels of nucleotide bases with strong Hydrogen Bonding (data not shown).

The transcription factor, CREB, is ubiquitously expressed in brain cells and is involved, among others, in learning and memory, anxiety, depression, and addiction [33]. A number of different signalling pathways culminate in the activation of CREB. These include pathways involving PKA, MAPK-activated ribosomal S6 kinases (RSKs), and calcium/calmodulin-dependent kinase IV (CaMKIV) [34]. Others such as CaMKII reduce CREB transcriptional activity [35]. Four genes have previously been found, by two independent microarray platforms, to be up-regulated in the ILS mouse cerebellum relative to the ISS cerebellum [22]. Of these, three were predicted by C4.5 as being CREB-dependent (Table 5). Similarly, three out of four genes up-regulated in the ISS cerebellum relative to the ILS cerebellum were predicted by the C4.5 scheme to be transcriptionally CREB-dependent (Table 6). Of a cross-section of genes up-regulated in the ILS cerebellum relative to ISS per the Affymetrix MOE430 platform [22], 64% were predicted by the C4.5 scheme to be transcriptionally CREB-dependent. Out of a similar cross-section up-regulated in the ISS cerebellum relative to the ILS cerebellum, 52% were predicted to be CREB-dependent. These indicate that CREB may be playing a central transcription-regulating role in the cerebellum in this ethanol sensitivity model.

## Conclusion

Taken together, the observations made suggest that, in the cerebellum, CREB plays a key role in ethanol sensitivity and presents the field with a central hypothesis that needs to be further tested. CREB's role in mediating a number of complex behaviours has been documented [33]. Events in the extended amygdala have long been associated with the reinforcing effects of addicting drugs [36]. It is evident that the cerebellum, though less well studied in this regard, is involved in addiction [13-15]. Since CREB's transcription regulating activity differs from cell type to cell type [37], pursuit of the implications of a key role for CREB in this addiction model's cerebellar molecular milieu would be both promising and instructive.

## Methods

A training dataset for ML was created out of twenty-three known targets of CREB transcriptional regulation [38,39], and twenty-three genes out of a set of twenty-eight (Table 7) whose transcription regulations are potentially CREB-independent. An independent set of twenty-one genes served as an evaluation set.

### "Potentially CREB-independent genes"

Nrf2 binds to CREB Binding Protein for enhanced transcription activating activity [25]. Cigarette Smoke (CS)-induced oxidative stress has been associated with the expression of Nrf2 transcription-dependent antioxidant and cytoprotective genes [40]. In experiments conducted by authors V.M and S.B., Nrf2 knockout and Wild-type mice were exposed to CS and Air. The genes listed in Table 7 were up-regulated in both groups, suggesting that their transcriptional regulation is Nrf2-independent (see "Oligonucleotide Microarray" below for further details on what constitutes "Nrf2-independent" genes). Furthermore, none of these genes is known specifically to be a target of CREB transcription regulation. Additionally, as depicted in Figure 3, these genes are distinguishable from those that are known targets for CREB transcription regulation.

#### CS Exposure

Mice of both genotypes were subjected to cigarette smoke exposure using a machine similar to the one used by [41]. The control groups were kept in a filtered air environment, and the experimental groups were subjected to CS for 5 hours by burning 2R4F reference cigarettes (2.45 mg nicotine per cigarette; Tobacco Research Institute, University of Kentucky), using a smoking machine (Model TE-10, Teague Enterprises). Details of the smoking protocol have been described previously [40]. Mice were fed AIN-76A diet (Harlan Teklad) and had access to water *ad libitum*; they were housed under controlled conditions (23 ± 2°C; 12-hour light/dark cycles). All experimental protocols conducted on the mice were performed in accordance with the standards established by the US Animal Welfare Acts, as set forth in NIH guidelines and in the Policy and Procedures Manual of the Johns Hopkins University Animal Care and Use Committee.

#### Oligonucleotide Microarray

Lungs were isolated after 5 hours of CS exposure. Total RNA from the lungs was extracted, using TRIZOL reagent (Invitrogen Corp.). The isolated RNA was hybridized to Murine Genome MOE 430 2.0 GeneChip arrays (Affymetrix, Santa Clara, CA) according to procedures described previously [40]. This array contains probes for detecting approximately 14,500 well-characterized genes and 4371 expressed sequence tags. Scanned output files were analyzed using Affymetrix GeneChip Operating Software version 1.3, and were independently normalized to an average intensity of 500. The data was further analyzed as described previously [42], by performing 9 pairwise comparisons for each group (Nrf2+/+, CS, n = 3, versus Nrf2+/+, air, n = 3, and Nrf2-/-, CS, n = 3, versus Nrf2-/-, air, n = 3). To limit the number of false positives, only those altered genes that showed more than a 1.5-fold change (FC) in magnitude and appeared in, at least, 6 of the 9 comparisons were selected. In addition, the Mann-Whitney pairwise comparison test was performed to rank the results by the significance (*P *≤ 0.05) of each identified change in gene expression. In identifying transcriptionally Nrf2-independent genes, only those genes which passed all of these criteria were selected. Further, only those genes that were differentially induced (or repressed) by CS to a similar extent in both genotypes, and having a FC ≥ 2.0 magnitude were considered to be independent of Nrf2's transcription regulating activity. This last dataset was combined with data from previously published work [40] (Genechip used was Murine U74A version 2) to arrive at a comprehensive "Nrf2-independent" gene set.

### Promoter Sequence Characteristics

**Figure 4:**
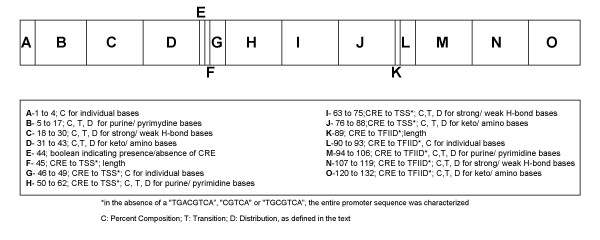
**Promoter Region Vectors**. Breakdown of the elements of the vector generated for each gene. For each gene, 132 numbers were generated from the promoter sequence. Blocks A through O are itemizations of the elements of the vector obtained for each gene.

Promoter sequences (1000 nucleotides upstream to 100 nucleotides downstream) corresponding to each gene was obtained from the cited database source [43]. For each promoter sequence, a vector of size 132, with elements characterizing features of the sequence (Figure 4) was generated using a Common Lisp [44] algorithm. The elements of the vector included a Boolean indicating whether or not the cAMP Response Element (CRE) was present, the number of nucleotide base pairs ("distance") between the CRE ("TGACGTCA", "CGTCA" or "TGCGTCA") and the Transcription Start Site (TSS), and the "distance" between the CRE and the TFIID bind site ("TATAGAA", "TATAAAA," "TATAG", or "TATA").

In addition to these, the three kinds of features of nucleotide sequences used were *Composition*, *Transition *and *Distribution *[23]. *Composition *is a reference to the proportions of nucleotide base types contributing to the promoter sequence make-up. *Transitions *represent the frequency with which specific nucleotide base types are followed or preceded, within the sequence, by other nucleotide base types. *Distribution *is a statement concerning the dissemination of specific nucleotide base types within portions of the sequence (or the entire sequence).

### Nucleotide Base Types

For the purpose of the sequence characterizations just described nucleotide bases were grouped based on whether they were purine or pyrimidine, the strength with which they form hydrogen bonds, and whether or not they were "keto" or "amino" (Table 1).

The breakdown of the elements of each vector (Figure 4) is as follows: percent Compositions for the individual nucleotide bases (positions 1 to 4); percent Compositions, Transitions, and Distributions for the Purine versus Pyrimidine base types (positions 5 – 17, consisting of two positions for Compositions, one for Transitions, and ten for Distributions); percent Compositions, Transitions, and Distributions for Strong versus Weak Hydrogen Bonding base types (positions 18 – 30, consisting of two positions for Compositions, one for Transitions, and ten for Distributions), percent Compositions, Transitions, and Distributions for "Keto" versus "Amino" base types (positions 31 – 43, consisting of two positions for Compositions, one for Transitions, and ten for Distributions). The presence or absence of a CRE was indicated by a "1" or a "0" respectively at position 44. The sub-sequence made up of the stretch of bases between the CRE and the TSS was characterized at positions 45 through 88. At position 45, the "distance" was stated. In the absence of a CRE, the entire promoter sequence was characterized in *lieu *of the sought sub-sequence. In other words, in the absence of a CRE as defined above, the "distance" was longer. Details for positions 46 through 48 were as follows: individual nucleotide base percent Compositions were indicated at positions 46 – 49; Purine versus Pyrimidine base type data were at positions 50 – 62; Strong versus Weak Hydrogen Bonding base type data were at positions 63 – 75; "Keto" versus "Amino" base type data were at positions 76 – 88. Correspondingly, the sub-sequence made up of the stretch of bases between the CRE and the TFIID bind site was similarly characterized at positions 89 through 132.

Four ML schemes were evaluated for their learning performance on the models created: a Decision Tree (J48, an implementation of the C4.5 algorithm), a Support Vector Machine (SVM), a Naïve Bayes classifier (NN) and a Multi-layered Perceptron (MLP), all available through the Weka ML workbench [45]. The C4.5 algorithm emerged as having the lowest predicted error rate (Figure 1). The decision tree (Additional File 2) used in evaluating the independent dataset is based on all the training data. After applying the Corrected Resampled t-test [46] to data generated following use of the leave-one-out technique with ten iterations for each fold, error rates for C4.5 were significantly (p = 0.05) lower than those of SVM and MLP (Table 2). The rates were lower relative to NN though not statistically significant (Table 2). The ROC curves (Figure 2) used as indicators of performance were also generated using the "CREB-regulated" class and the default Weka ML workbench. The threshold modifications that constituted the basis of the ROC curves have been detailed in Additional File 1.

Subsequently a set of genes whose expressions have been previously determined [22] to be increased in ILS or ISS cerebella was identified and the CREB regulation status of each member predicted using the ML scheme C4.5.

Exploratory Data Analysis (EDA) techniques [47] were also used to characterize the vector set. Specifically, boxplots [48] were used to capture the distribution's central tendency (median), spread (fourth-spread), skewness (based on the relative positions of the median, lower fourth and upper fourth), tail length as well as outliers (Figure 3). The statistical environment used to implement the EDA aspects of the study was R [49].

## Authors' contributions

GKA conceived the study, conducted the computational experiments, and participated in writing the manuscript. VM participated in the conduct of experiments and data analyses resulting in the identification of Nrf2-independent genes. He also wrote portions of the manuscript. SB participated in the experimental design, participated in the conduct of experiments and data analyses resulting in the identification of Nrf2-independent genes. He also participated in drafting portions of the manuscript. All authors read and approved the final manuscript.

## Supplementary Material

Additional File 1 Table detailing threshold modifications that generated the data for the ROC curves (Figure 2). This was done using the "CREB-regulated" class and the default Weka ML workbench. For each learning scheme, the thresholds are a list of predicted probabilities of the "CREB-regulated" class. The predicted probabilities are calculated by the respective learning schemes, and depend on the numbers of True Positives, False Negatives, False Positives, and True Negatives. As an example, at a threshold of 0.33, the C4.5 scheme classifies an instance as "CREB-regulated" only if the calculated predicted probability of "CREB-regulated" is greater than 0.33. At each threshold, there was a False Positive rate and a corresponding True Positive rate; these then were used to plot the ROC curves. The ranked list of points was classifier-dependent. Click here for file

Additional File 2 This is a top-down depiction of the decision tree generated based on *all *the training data. The oval nodes represent the attributes chosen by the C4.5 algorithm based on *information gain*. The root node, i.e. the "distance" between the CRE and the transcription start site (as detailed in Fig 4 and the Methods section), is a principal attribute determined by the C4.5 algorithm as useful for distinguishing between the two classes. The edges represent the cut-offs in value of the attribute represented in the originating oval node. The rectangular nodes are the classifications arrived at: "YES" represents "CREB-regulated"; "NO" represents "NOT CREB-regulated". (In the rectangular nodes, the numbers within brackets represent "*number of correctly classified instances" *or "*number of correctly classified*/*number of incorrectly classified instances"*). Click here for file

## Figures and Tables

**Table 1:** Nucleotide base groupings used

**GROUP**	**MEMBERS**
Purine	A, G
Pyrimidine	C, T
Strong Hydrogen Bonding	C, G
Weak Hydrogen Bonding	A, T
Keto	T, G
Amino	A, C

**Table 2:** Performance of learning schemes following 460 runs**

	**C4.5**	**SVM**	**NN**	**MLP**
**PERCENT CORRECT**	69.57 (46.06)	50.00 (50.05) *	58.70 (49.29)	55.00 (49.80)
**MEAN ABSOLUTE ERROR**	0.18 (0.28)	0.33 (0.11) *	0.28 (0.33)	0.30 (0.30) *
**RELATIVE ABSOLUTE ERROR**	52.51 (80.99)	96.00 (32.03) *	79.30 (94.63)	85.53 (86.77) *
**ROOT MEAN SQUARED ERROR**	0.22 (0.34)	0.41 (0.14) *	0.34 (0.40)	0.36 (0.37) *
**ROOT RELATIVE SQUARED ERROR**	53.54 (82.59)	97.90 (32.67) *	80.86 (96.50)	87.11 (88.44) *

The standard deviation of each attribute evaluated is located in brackets

**Leave-one-out technique, i.e. 46-fold cross-validation, performed with ten iterations each

*Use of Corrected Resampled T-test [44]; difference from corresponding C4.5 value is statistically significant (p = 0.05, two tailed)

**Table 3:** Area under ROC curves, two-class model.

	**INDEPENDENT EVALUATION SET**	**TEN-FOLD CROSS VALIDATION**	**LEAVE-ONE-OUT**
**C4.5**	0.8563	0.7722	0.7883
**NN**	0.7875	0.5936	0.6352
**SVM**	0.9063	0.5217	0.5
**MLP**	0.85	0.6711	0.5577

**Table 4:** Evaluation of two-class model: C4.5 predictions on an independent set of genes of known CREB regulation status**

**GENE SYMBOL**	**C4.5 PREDICTION**	**CONFIDENCE LEVEL**	**ACTUAL STATUS**
Pcna	CREB-REGULATED	1	CREB-REGULATED
Pdyn	CREB-REGULATED	1	CREB-REGULATED
Penk1	CREB-REGULATED	1	CREB-REGULATED
Ptgs2	CREB-REGULATED	1	CREB-REGULATED
Pck1	NOT-CREB-REGULATED*	0.8	CREB-REGULATED
Ppargc1a	CREB-REGULATED	1	CREB-REGULATED
Muc5b	CREB-REGULATED	1	CREB-REGULATED
Rb1	CREB-REGULATED	1	CREB-REGULATED
Sst	NOT-CREB-REGULATED*	0.8	CREB-REGULATED
Aanat	CREB-REGULATED	1	CREB-REGULATED
Sod2	CREB-REGULATED	1	CREB-REGULATED
Sms	CREB-REGULATED	1	CREB-REGULATED
Tnp1	CREB-REGULATED	1	CREB-REGULATED
Th	NOT-CREB-REGULATED*	1	CREB-REGULATED
Vip	CREB-REGULATED	1	CREB-REGULATED
Slc18a2	CREB-REGULATED	1	CREB-REGULATED
Kif1b	CREB-REGULATED	1	NOT-CREB-REGULATED*
Tcf21	NOT-CREB-REGULATED*	1	NOT-CREB-REGULATED*
Wisp2	NOT-CREB-REGULATED*	1	NOT-CREB-REGULATED*
Ms4a4c	NOT-CREB-REGULATED*	1	NOT-CREB-REGULATED*
Lrat	NOT-CREB-REGULATED*	1	NOT-CREB-REGULATED*

*"Potentially CREB-independent" genes as defined under the Methods section.

**This follows training with a set of 46 genes of known status: twenty-three "CREB regulated" and twenty-three "Not CREB regulated" instances

**Table 5:** C4.5 two-class model predictions for up-regulated genes (cross-validated between MOE430 and NIA15k platforms) in ILS mouse cerebellum

**GENE SYMBOL**	**C4.5 PREDICTION**	**CONFIDENCE LEVEL**
Chchd4	CREB-REGULATED	1
Sca1	CREB-REGULATED	1
Myo1d	NOT-CREB-REGULATED*	1
6430706D22Rik	CREB-REGULATED	1

*"Potentially CREB-independent" genes as defined under the Methods section.

**Table 6:** C4.5 two-class model predictions for up-regulated genes (cross-validated between MOE430 and NIA15k platforms) in ISS mouse cerebellum

**GENE SYMBOL**	**C4.5 PREDICTION**	**CONFIDENCE LEVEL**
Cap1	CREB-REGULATED	1
D7Rp2e	NOT-CREB-REGULATED*	1
Ftl1	CREB-REGULATED	1
Gnb1	CREB-REGULATED	1

*"Potentially CREB-independent" genes as defined under the Methods section.

**Table 7:** List of "potentially CREB-independent" genes

**Gene Title**	**Gene Symbol**
FK 506 binding protein 5	Fkbp5
cyclin-dependent kinase inhibitor 1A (P21)	Cdkn1a
growth arrest and DNA-damage-inducible 45 gamma	Gadd45g
angiopoietin-like 4	Angptl4
adrenomedullin	Adm
DNA-damage-inducible transcript 4	Ddit4
chromodomain helicase DNA binding protein 1	Chd1
sema domain, immunoglobulin domain (Ig), and GPI membrane anchor,	Sema7a
chloride channel calcium activated 1	Clca1
quiescin Q6	Qscn6
sestrin 1	Sesn1
UDP-N-acetyl-alpha-D-galactosamine:polypeptide N-	Galntl2
breast carcinoma amplified sequence 3	Bcas3
membrane-associated protein 17	Map17
discs, large homolog-associated protein 4 (Drosophila)	Dlgap4
glucosidase 1	Gcs1
protein related to DAN and cerberus	Prdc
thymidylate kinase family LPS-inducible member	Tyki
histidine decarboxylase	Hdc
sorting nexin 16	Snx16
androgen-induced proliferation inhibitor	Aprin
acylphosphatase 1, erythrocyte (common) type	Acyp1
intersectin (SH3 domain protein 1A)	Itsn
kinesin family member 1B	Kif1b
transcription factor 21	Tcf21
WNT1 inducible signaling pathway protein 2	Wisp2
membrane-spanning 4-domains, subfamily A, member 4C	Ms4a4c
lecithin-retinol acyltransferase (phosphatidylcholine-retinol-O-acyltransferase)	Lrat
