# Supplementary figures and images for: Ethanol sensitivity: a central role for CREB transcription regulation in the cerebellum
Source: BMC Genomics. 2006 Dec 5;7:308. doi: 10.1186/1471-2164-7-308 (PMC1698922; doi:10.1186/1471-2164-7-308)

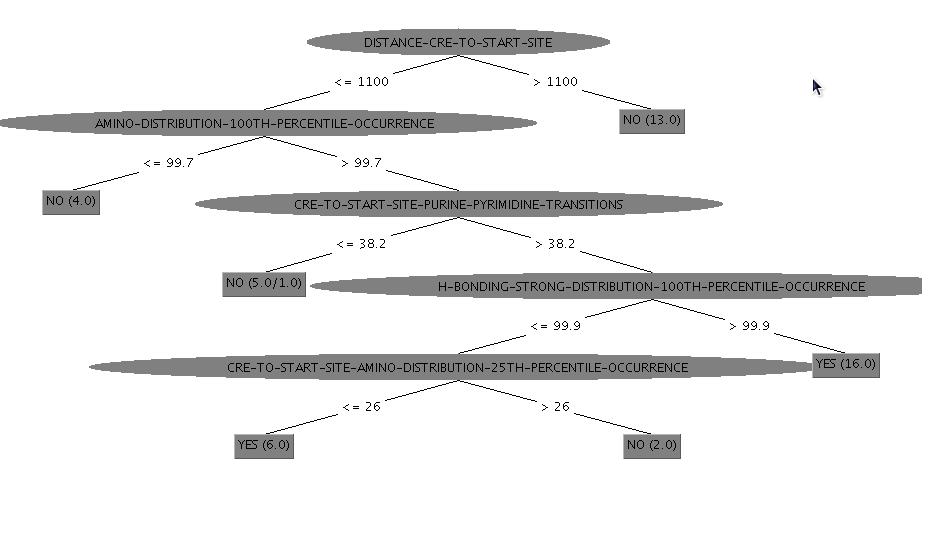

Supplement: Additional File 2 — This is a top-down depiction of the decision tree generated based on all the training data. The oval nodes represent the attributes chosen by the C4.5 algorithm based on information gain. The root node, i.e. the "distance" between the CRE and the transcription start site (as detailed in Fig 4 and the Methods section), is a principal attribute determined by the C4.5 algorithm as useful for distinguishing between the two classes. The edges represent the cut-offs in value of the attribute represented in the originating oval node. The rectangular nodes are the classifications arrived at: "YES" represents "CREB-regulated"; "NO" represents "NOT CREB-regulated". (In the rectangular nodes, the numbers within brackets represent "number of correctly classified instances" or "number of correctly classified/number of incorrectly classified instances"). [file 1471-2164-7-308-S2.jpeg]
